# Healthy Human Fecal Microbiota Transplantation into Mice Attenuates MPTP-Induced Neurotoxicity *via* AMPK/SOD2 Pathway

**DOI:** 10.14336/AD.2023.0309

**Published:** 2023-12-01

**Authors:** Zhenchao Xie, Mahui Zhang, Yuqi Luo, Dana Jin, Xingfang Guo, Wanlin Yang, Jialing Zheng, Hongfei Zhang, Lu Zhang, Chao Deng, Wenhua Zheng, Eng-King Tan, Kunlin Jin, Shuzhen Zhu, Qing Wang

**Affiliations:** ^1^Department of Neurology, Zhujiang Hospital of Southern Medical University, Guangzhou, Guangdong, China.; ^2^College of Biological Science, University of California, Davis, CA 95616, USA.; ^3^Department of Anaesthesiology, Zhujiang Hospital of Southern Medical University, Guangdong, China.; ^4^Key Laboratory of Functional Proteomics of Guangdong Province, Key Laboratory of Mental Health of the Ministry of Education, School of Basic Medical Sciences, Southern Medical University, Guangdong, China.; ^5^School of Medical, Indigenous and Health Sciences, and Molecular Horizons, University of Wollongong, Wollongong, Australia.; ^6^Centre of Reproduction, Development & Aging and Institute of Translation Medicine, Faculty of Health Sciences, University of Macau, Avenida de Universidade, Macau, China.; ^7^Department of Neurology, National Neuroscience Institute, Singapore General Hospital, Singapore.; ^8^Department of Pharmacology and Neuroscience, University of North Texas Health Science Center, Fort Worth, TX 76107, USA

**Keywords:** neuroinflammation, parkinson's disease, AMPK/SOD2, gut microbiota, pericytes, fecal microbiota transplantation

## Abstract

Increasing evidence has shown that gut dysbacteriosis may play a crucial role in neuroinflammation in Parkinson's disease (PD). However, the specific mechanisms that link gut microbiota to PD remain unexplored. Given the critical roles of blood-brain barrier (BBB) dysfunction and mitochondrial dysfunction in the development of PD, we aimed to evaluate the interactions among the gut microbiota, BBB, and mitochondrial resistance to oxidation and inflammation in PD. We investigated the effects of fecal microbiota transplantation (FMT) on the physiopathology of 1-methyl-4-phenyl-1,2,3,6-tetrahydropyridine (MPTP)-treated mice. The aim was to explore the role of fecal microbiota from PD patients and healthy human controls in neuroinflammation, BBB components, and mitochondrial antioxidative capacity via the AMPK/SOD2 pathway. Compared to control mice, MPTP-treated mice exhibited elevated levels of Desulfovibrio, whereas mice given FMT from PD patients exhibited enriched levels of Akkermansia and mice given FMT from healthy humans showed no significant alterations in gut microbiota. Strikingly, FMT from PD patients to MPTP-treated mice significantly aggravated motor impairments, dopaminergic neurodegeneration, nigrostriatal glial activation and colonic inflammation, and inhibited the AMPK/SOD2 signaling pathway. However, FMT from healthy human controls greatly improved the aforementioned MPTP-caused effects. Surprisingly, the MPTP-treated mice displayed a significant loss in nigrostriatal pericytes, which was restored by FMT from healthy human controls. Our findings demonstrate that FMT from healthy human controls can correct gut dysbacteriosis and ameliorate neurodegeneration in the MPTP-induced PD mouse model by suppressing microgliosis and astrogliosis, ameliorating mitochondrial impairments via the AMPK/SOD2 pathway, and restoring the loss of nigrostriatal pericytes and BBB integrity. These findings raise the possibility that the alteration in the human gut microbiota may be a risk factor for PD and provide evidence for potential application of FMT in PD preclinical treatment.

Parkinson’s disease (PD) is the second leading neurodegenerative disease in the world, impacting more than 1.5% of people aged over 65 years, and its prevalence is expected to double by 2030 [1]. Less than 10% of PD cases are attributed to inherited genetic elements, while the majority of PD cases are considered idiopathic or sporadic [2]. In addition to genetic predisposition, multiple factors, such as aging and environmental factors, play crucial roles in the etiology of PD [3]. Neuro-inflammation, autophagy-lysosomal disorders, ubiquitin-proteasomal impairment, mitochondrial dysfunction, and oxidative stress are widely recognized as the main factors underlying the pathophysiology of PD [4, 5]. Aging is driven by oxidative stress to macromolecules, initially proposed by Harman et al. [6], and subsequently expanded to recognize mitochondria as the primary source of free radicals and targets of oxidation [7]. In the last two decades, increasing evidence has confirmed that mitochondrial defects and oxidative stress increase with age [8]. Neurotoxicants, such as 1-methyl-4-phenyl-1,2,3,6-tetrahydropyridine (MPTP), paraquat, rotenone and 6-hydroxydopamine (6-OHDA), are commonly used to induce experimental PD models by disrupting mitochondria and increasing oxidative stress [9]. Moreover, mutations in PD-associated genes, such as SNCA, PINK1, PRKN, LRRK2, and DJ-1, are also linked to mitochondrial dysfunction [10]. Thus, mitochondrial malfunction is increasingly considered a key determinant of dopaminergic neurodegeneration, serving as an overlapping risk factor for both sporadic and familial cases [11].

In addition to the typical movement disorders, PD patients also suffer from various nonmotor manifestations, such as constipation [12, 13]. Braak et al. theorized that the pathology of PD might begin in the gut and gradually progress to the brain [14], which was partially supported by subsequent research [15]. However, the underlying cause of gut pathology in PD remains unclear. Recent evidence has demonstrated that gut dysbacteriosis is prevalent among PD patients and may be related to motor symptoms [16], suggesting that gut microbiota may be crucial in the development of PD. Sampson et al. demonstrated that fecal microbiota transplantation (FMT) from PD patients to mice led to the aggravation of motor deficits via the activation of microglia, providing experimental evidence for the role of gut microbiota in PD [17]. Furthermore, recent studies revealed that FMT from normal control mice into neurotoxin-induced PD mice could protect dopaminergic neurons by suppressing the TLR4 inflammatory pathway [18, 19].

Given that neuroinflammation does not represent the primary pathophysiology of PD and mitochondrial dysfunction is a hallmark of PD, we aim to investigate the potential effects of the gut microbiota on the brain via regulation of mitochondrial function beyond inflammation. Interestingly, a recent study showed that sleep deprivation caused mice and flies death through the accumulation of reactive oxygen species (ROS) in the gut [20], suggesting a possible link between gut microbiota and mitochondrial function. Emerging data have revealed that gut microbiota may regulate mitochondrial metabolism [21] and inflammation [22] by interacting with mitochondria. Oxidative stress is closely correlated with the neuropathogenesis of PD, and SOD2 is a mitochondria-localized antioxidant enzyme [23]. Intriguingly, intestinal AMPK, which can directly or indirectly interact with SOD2 [24, 25], was discovered to be modulated by microbiota [26]. However, it remains unclear how the gut microbiota affects the brain. Owing to its stringent impermeability to most molecules, the blood-brain barrier (BBB) maintains the subtle homeostasis of the central nervous system and mediates dialog between the central nervous system and the periphery by selectively transporting soluble molecules [27]. The BBB is made up of numerous layers, including the glycocalyx, endothelial cells, the basement membrane (which encompasses pericytes), and astrocytic end-feet [28]. Furthermore, dysfunction of the BBB may contribute to the onset and progression of PD [29]. Pericytes play an essential role in maintaining the integrity of the BBB [30], and loss of pericytes may increase BBB permeability, allowing harmful intestinal substances to enter the brain and damage neurons.

Based on the common occurrence of gut symptoms and gut dysbacteriosis in PD patients and the growing evidence of the interaction between inflammation and mitochondria in the brain, we hypothesized that FMT from PD patients or healthy controls could regulate the hallmark motor deficits in MPTP-treated mice by modulating mitochondrial antioxidative capacity and pericyte loss. Herein, we found that gut dysbacteriosis is sufficient to promote disease symptoms. Specifically, FMT from PD patients significantly impairs motor function in MPTP-treated mice, while microbiota from healthy controls may prevent these effects, likely by regulating inflammation, mitochondrial antioxidative capacity and pericytes. These results provide important insights into the potential application of FMT in the PD pre-clinical treatment.

## MATERIALS AND METHODS

### Animals

Ten-week-old male C57BL/6J mice (26 ± 2 g) were obtained from Guangdong Medical Laboratory Animal Center (Foshan, China). All mice were raised in the same facility under the same 12-hour light/dark cycle and controlled conditions (temperature 21 ± 1 °C and humidity 55 ± 5%) with unlimited access to food and water. All experimental procedures were approved by the Animal Ethics Committee of Zhujiang Hospital, Southern Medical University (No: LAEC-2021-045).

The mice were randomly divided into four groups: control group, (control; n = 12), given saline by intraperitoneal injection (i.p.) for 5 days followed by oral administration of PBS containing 20% sterile glycerol (0.2 ml)for 10 days; MPTP-treated group (MPTP; n = 12), given MPTP (i.p.) (30 mg/kg) for 5 days followed by oral administration of PBS containing 20% sterile glycerol (0.2 ml) for 10 days; MPTP-injected plus FMT from PD patients group (MPTP + PD FMT; n = 12), given MPTP (i.p.) for 5 days followed by FMT from PD patients for 10 days; and MPTP-injected plus FMT from healthy human controls group (MPTP + HC FMT; n = 12), given MPTP (i.p.) for 5 days followed by FMT from healthy human controls for 10 days.

### Human Donors and Criteria

This study was conducted in accordance with the Helsinki Declaration, revised in 1975, and the National Institutes of Health Policy and Guidelines for Human Subjects, issued in 1999, and was approved by the Ethics Committee of Zhujiang Hospital of Southern Medical University. Informed consent was obtained from all participants, and the study enrolled only those individuals with PD who met the Movement Disorder Society Clinical Diagnostic Criteria for idiopathic PD [31]. The exclusion criteria for PD participants were as follows: reluctant to provide feces; use of antibiotics or probiotics within 12 weeks of feces sampling; bowel diseases; use of nonsteroidal anti-inflammatory drugs; unstable neurological or psychiatric illness; and secondary or atypical Parkinsonism. The healthy human controls were as closely matched to the PD donors as possible, with the following inclusion criteria: normal physical and blood examination, no bowel diseases or neurodegenerative conditions, and no use of probiotics or antibiotics 12 weeks prior to feces collection [17]. For fecal microbiota donor, we recruited six pairs of participants [17]. All experimental procedures involving humans were approved by the Human Ethics Committee of Zhujiang Hospital, Southern Medical University (No: 2022-KY-242-01).

### Subacute MPTP-induced PD mouse model and FMT treatment

For the MPTP injection, 90 mg MPTP (Sigma-Aldrich, German) was dissolved in 30 ml of sterile saline. Mice received intraperitoneal (i.p.) injection of MPTP (30 mg/kg) once daily for 5 days, with the control mice receiving saline injections [32]. For FMT, fresh feces were collected from PD patients or healthy human controls, and 1 g of stool was dissolved in 15 ml bacteria-free oxygen-free phosphate-buffered saline (PBS). The suspension was centrifuged at 3000 g and 4 °C for 5 min, and 12 ml of supernatant was added to 3 ml of bacteria-free glycerol to reach a final concentration of 20% and then kept at -80 °C for 1-8 weeks before use. Thawing was performed in an ice bath before FMT. The gut microbiota was transplanted into the mice by gavage with 0.2 ml per mouse administered daily for 10 days [33].

### Behavioral tests

Pole test: Each mouse was positioned on top of a 55 cm long, 1 cm diameter pole, and the time needed to arrive at the bottom was tested. The trial began on the day following the last treatment. Three trials with a one-hour gap were performed for each mouse, and the average time was taken [34].

Rotarod test: The rotarod test was used to assess motor coordination. Before recording, the mice received training 3 times on the rotating rod at 15 rpm. For recording, the mice were placed facing forward on the rod, which was rotated at a speed that increased from 4 to 40 rpm for 5 min. The time each mouse stayed on the rod before falling was measured. The test was repeated 3 times with a 1 h interval [34].

### Western blotting

After anaesthetization with isoflurane, the mice were subjected to systemic circulation perfusion with ice-cold PBS, after which fresh brain and gastrointestinal tissue were dissected swiftly and frozen in liquid nitrogen before storing them at -80 °C. Total protein was extracted from the tissues using a homogenizer and radioimmuno-precipitation assay (RIPA) buffer with a phosphatase inhibitor mixture, 1% phenylmethanesulfonyl fluoride, and protease inhibitor cocktail. After centrifugation at 12,000 × g and 4 °C for 10 min, the supernatant was taken to obtain the total protein. The protein concentration was quantified with a BCA Protein Assay Kit, and 25 µg of protein was separated by electrophoresis on an SDS-polyacrylamide gel and transferred to a poly (vinylidene fluoride) membrane. Each membrane was blocked with 5% bovine serum albumin for 1 hour at room temperature, followed by incubation overnight at 4 °C with primary antibodies, including rabbit anti-tyrosine hydroxylase (TH) antibody (1:1000, #58844, CST), rabbit anti-interleukin-1β (IL-1β) antibody (1:1000, #12703, CST), rabbit anti-phosphorylated-AMPK (p-AMPK) antibody (1:1000, #2537, CST), rabbit anti-SOD2 (SOD2) antibody (1:1000, #13141, CST), and rabbit anti-β-actin antibody (1:1000, #4967, CST). The membranes were then incubated for 1 hour at room temperature with HRP-conjugated anti-rabbit IgG secondary antibody (1:10000, #7074, CST). A Bio-Rad chemiluminescence system (Bio-Rad, USA) was used to detect the blots, and Fiji ImageJ software was used to measure the band densities.

### Hematoxylin and eosin staining and histologic scoring

In brief, after systemic circulation perfusion with ice-cold PBS followed by ice-cold 4% paraformaldehyde (PFA), the brains and guts of mice were collected and then fixed in 4% PFA for no more than 24 h. The samples were then processed using a Sakura Finetek tissue processor (Tissue-Tek® VIP™ 5Jr) as follows: (1) 50% ethanol for 30 min; (2) 75% ethanol for 40 min; (3) 85% ethanol for 50 min; (4) 95% ethanol for 60 min; (5) 100% ethanol for 60 min; (6) 100% ethanol I for 50 min; (7) 100% ethanol II for 40 min; (8) xylene I for 40 min; (9) xylene II for 50 min; (10) xylene III for 60 min; (11) paraffin I for 40 min; (12) paraffin II for 50 min; and (13) paraffin III for 60 min. Subsequently, the tissues were embedded in paraffin and sliced into 6-μm-thick transverse sections using a rotary microtome (Leica, German) [35]. After baking at 65°C for 1 hour, the sections underwent deparaffinization in xylene I, II, and III for 10 min each, followed by rehydration in a graded series of ethanol solutions (100%, 95%, 85%, and 75% ethanol) for 5 min each. After 3 min of hematoxylin staining, the sections were immersed in eosin for 30 s, and dehydrated using a series of ethanol solutions (75%, 85%, 95%, and 100% ethanol) for 30 seconds, 1 min, 3 min, and 3 min, respectively, and finally cleared in xylene and mounted with neutral resins [36]. Images were taken with a microscope (Leica, German). Histologic scoring was performed as the following criteria: 0, sporadic inflammatory cells in the lamina propria; 1, increased inflammatory cells infiltration into the lamina propria; 2, the confluence of inflammatory cells extending into the submucosa; 3, transmural inflammatory cell infiltration [37].

### Immunohistochemical and immunofluorescence staining

For immunohistochemical staining, followed by baking, deparaffinization, and rehydration, sections underwent heat-induced epitope retrieval and were immersed in 10 mM Na citrate buffer (pH 6.0) in a microwave oven. Endogenous peroxidase activity was blocked by incubating with 3% hydrogen peroxide solution for 15 min at room temperature, and the sections were then blocked with normal serum from the same species as that of the secondary antibodies for 15 min at room temperature. The following antibodies were applied at 4 °C overnight: rabbit anti-tyrosine hydroxylase (TH) antibody (1:300, #58844, CST), rabbit anti-Iba-1 antibody (1:300, #17198, CST), rabbit anti-GFAP antibody (1:300, #80788, CST), and rabbit anti-phosphorylated-AMPK (p-AMPK) antibody (1:200, #2537, CST). Biotin-conjugated goat-anti-rabbit IgG secondary antibody (1:1000, ab97049, Abcam) was applied for 30 min at room temperature. The sections were washed three times with PBS for 3 min each and incubated in streptavidin-conjugated HRP for 15 min at room temperature. 3,3'-Diaminobenzidine tetrahydrochloride was applied for 3 min at room temperature. Finally, the sections were dehydrated in 75%, 85%, 95%, and 100% ethanol for 3 min each, cleaned in xylene I, II, and III for 10 min each, and mounted with neutral resin. Of note, to determine the specificity of the primary antibody, we employed the isotype control of the primary antibody as a negative control. The isotype control antibodies were selected to match the host species, Ig class, or Ig subclass of the primary antibody. They (rabbit IgG, #3900, CST, USA; goat IgG, B900630, Proteintech, China) were applied at the same dilution ratio, incubation time, and solvent as the other primary antibodies. To further identify any potential non-specific staining by the tissue sections, we also utilized a control with only the secondary antibody. Images were taken with a microscope (Leica, German), and Fiji ImageJ software was used for quantitative analysis.

For immunofluorescence staining, heat-induced epitope retrieval was carried out in 10 mM Tris-EDTA (pH 9.0). The following primary antibodies were used: rabbit anti-GFAP (1:100, #80788, CST), rabbit anti-Iba-1 (1:300, #17198, CST), rabbit anti-ZO-1 (1:1000, ab221547, Abcam), rabbit anti-CD13 (1:100, #32720, CST), goat anti-PDGFRβ (1:200, AF1042, R&D), and goat anti-CD31 (1:200, GB13063, Servicebio). The samples were incubated with secondary antibodies (donkey anti-rabbit Alexa-488 IgG,1:500, ab150073, Abcam; donkey anti-goat Alexa-555 IgG, 1:500, ab150130, Abcam) for 1 hour at room temperature in the dark and then mounted using Aqueous Mounting Medium (Abcam, UK). Images were captured using an inverted fluorescence microscope (Nikon, Japan). The number of positive cells was counted by hand. Fiji ImageJ software was used to calculate the positive signal optical density.

### 16S ribosomal DNA (rDNA) sequencing of fecal samples

Fresh fecal samples from mice (n = 7 in each group) were collected ten days after gavage administration of fecal microbiota or glycerin. The total DNA was extracted using an E.Z.N.A. ®Stool DNA Kit (D4015, Omega, Inc., USA) according to the manufacturer's instructions. Primers 341 F (5′- CCTACGGGNGGCWGCAG-3′) and 805 R (5′- GACTACHVGGGTAT CTAATCC-3′) tagged with barcode sequences were used to amplify the V3-V4 region of 16S rDNA [38]. The PCR amplification mixture contained 25 ng of template DNA, 12.5 mL of PCR Premix, 2.5 mL of each primer, and DEPC water to control the total volume, and was mixed to a total reaction solution of 25 mL. After identification via 2% agarose gel electrophoresis, the purified PCR products were quantified using a Qubit fluorometer (Invitrogen, USA). The amplicon pools were provided for sequence analysis, and the size and quantity of the amplicon libraries were defined using an Agilent 2100 Bioanalyzer (Agilent, USA) and an Illumina Library Quantification Kit (Kapa Biosciences, Woburn, MA, USA). LC-BioTechnology Co., Ltd. (Hang Zhou, Zhejiang Province, China) sequenced the samples on an Illumina NovaSeq platform in accordance with the manufacturer’s recommendations. We analyzed not only the relative abundance of microbiota at different taxonomic levels but also alpha and beta diversity via the QIIME2 process, and the feature sequences were annotated with the SILVA database [39].

### Statistical analysis

Statistical analysis was performed using Graph Pad Prism software. One-way ANOVA with the Tukey test for multiple comparisons was used for data with normal distribution, while Kruskal-Wallis test with the Dunn’s test for multiple comparisons was used when the data did not follow a normal distribution. Results are presented as means ± SEM (standard error of the mean) for data with normal distribution, otherwise as medians (IQR) (interquartile range) for data with non-normal distribution. The statistical significance threshold was set at *P* < 0.05, with significance levels indicated as *P < 0.05, **P < 0.01, ***P < 0.001, ****P < 0.0001. Graphs were prepared using Graph Pad Prism version 8.0 (Graph Pad, Inc., La Jolla, USA) except that graphs concerning 16S rDNA sequencing were drawn using the R package (v3.5.2).

**Figure 1.**
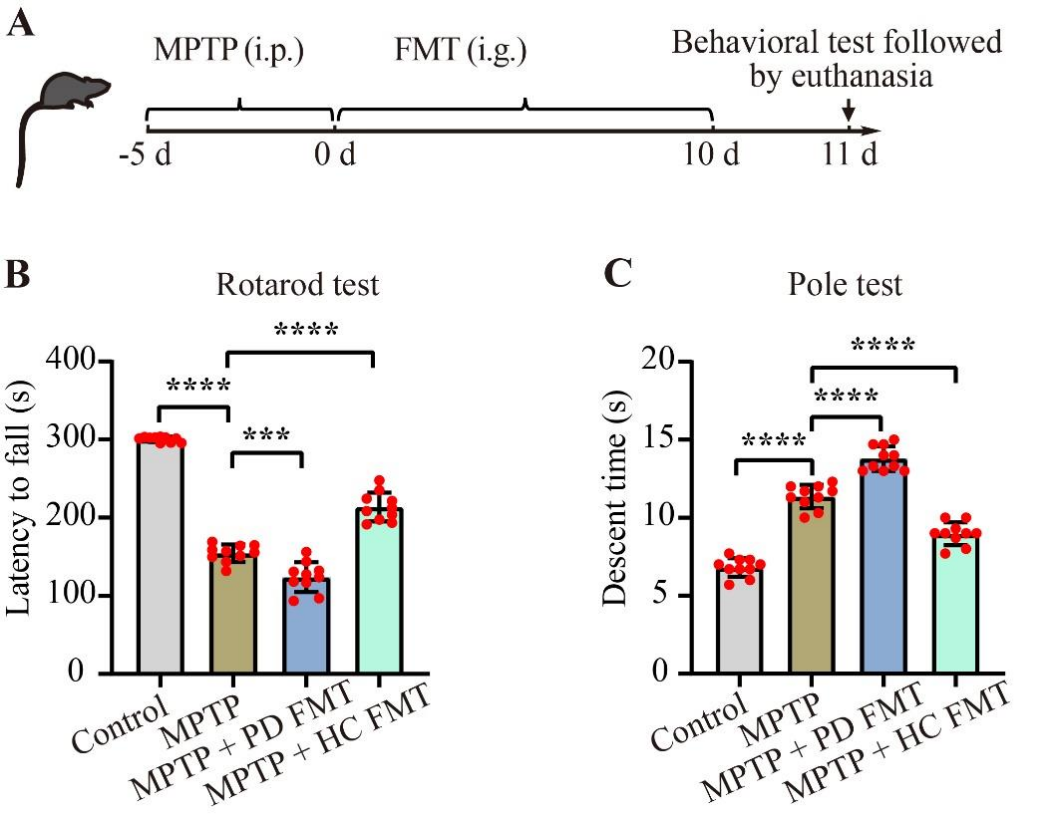
FMT from healthy human controls alleviated motor deficits in MPTP-treated mice. The experimental timeline for this study is depicted in (A). To assess motor function, all mice were given the rotarod test (B) and pole test (C) the day after the final FMT. Motor deficits were found in MPTP-treated mice, while FMT from PD patients significantly aggravated the deficits. However, FMT from healthy human controls significantly improved the motor deficits. Statistical analyses were performed using one-way ANOVA followed by Tukey test for multiple comparisons, with n = 10, and error bars indicating mean ± SEM. The significance levels were ***P < 0.01, ****P < 0.0001 (*P* values were adjusted with 6 tests). Abbreviations used are i.p. for intraperitoneal, i.g. for intragastric, and s for seconds.

**Figure 2.**
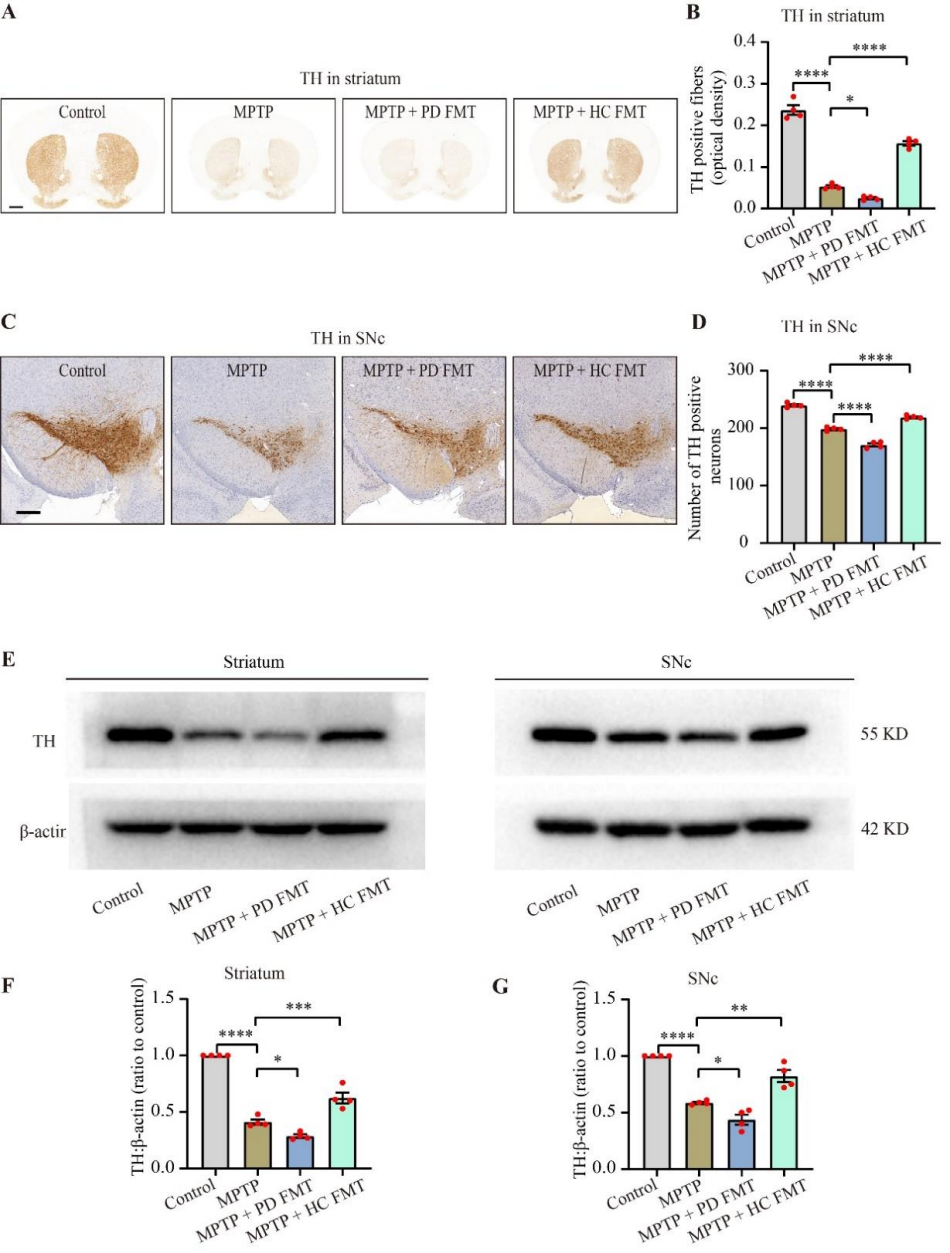
Fecal microbiota from healthy humans slowed down the MPTP-induced dopaminergic neurodegeneration. Immunohistochemistry was used to analyze TH^+^ fibers in the striatum (A) and TH^+^ neurons in the SNc (C), with scale bars of 1000 µm (A) and 100 µm (C). Quantitative analysis of the TH^+^ fibers area in the striatum (B) and the number of TH^+^ neurons in the SNc (D) showed that FMT from PD patients accelerated the reduction in dopaminergic neurons and fibers in MPTP-treated mice, while FMT from healthy human controls rescued dopaminergic neurodegeneration. Western blot analysis of TH expression in both the striatum and SNc (E) confirmed the immunohistochemistry results. A significantly lower TH expression was observed in both the striatum (F) and SNc (G) in the MPTP + PD FMT group compared to the MPTP group. Conversely, the MPTP + HC FMT group showed higher TH expression than both the MPTP and MPTP + PD groups. Multiple comparisons were performed using one-way ANOVA followed by Tukey test, n = 4, and error bars indicate mean ± SEM. Statistical significance was denoted by * *P* < 0.05, ** *P* < 0.01, *** *P* < 0.001, and ****P < 0.0001 (*P* values were adjusted with 6 tests).

## RESULTS

### FMT from healthy human controls alleviated motor deficits in MPTP-induced PD mice

Before FMT, the control group mice received saline (i.p.) treatment for 5 days, while other mice were treated with MPTP (i.p.) for 5 days. Subsequently, mice in the MPTP + PD FMT group and MPTP + HC FMT group underwent ten days of FMT from PD patients or healthy human controls, respectively. The control group and MPTP group mice were administered PBS plus 20% glycerol by gavage for 10 days. All mice underwent behavioral testing the day following their last administration and were then euthanized (Fig. 1A). Motor deficits commonly occur in PD mouse models. To investigate whether gut microbiota from PD patients or healthy human controls can affect the motor function of MPTP-induced PD mice, we used rotarod test and pole test to assess motor coordination and bradykinesia, respectively. Compared to the control mice, the MPTP-treated mice exhibited a significantly shorter latency to fall from the rotatory rod (*P* < 0.0001, Fig. 1B), and a noticeably longer time to descend from the pole (*P* < 0.0001, Fig. 1C). Interestingly, compared to the MPTP group, the MPTP + PD FMT group presented a shorter latency time in the rotarod test (*P* < 0.001, Fig. 1B) and a longer descent time in the pole test (*P* < 0.0001, Fig. 1C), while the MPTP + HC FMT group presented a longer latency to fall (*P* < 0.0001, Fig. 1B) and a shorter descent time (*P* < 0.0001, Fig. 1C). These results indicated that the gut microbiota from PD patients might aggravate motor deficits in MPTP-induced PD mice, while FMT from healthy human controls might enhance the motor performance of MPTP-treated mice.

### FMT from healthy human controls slowed down the dopaminergic neurodegeneration induced by MPTP

PD is characterized by a significant loss of dopaminergic neurons, which are marked by the high expression of tyrosine hydroxylase (TH), the rate-limiting enzyme in dopamine biosynthesis [40]. To examine the effect of gut microbiota from PD patients or healthy human controls on dopaminergic neuron survival in the substantia nigra pars compacta (SNc) of MPTP-treated mice, we next simultaneously analyzed TH expression in the SNc and striatum via immunohistochemistry and western blotting. Immunohistochemistry showed that MPTP treatment significantly reduced TH^+^ fibers in the striatum (*P* < 0.0001, Fig. 2B) and TH^+^ somata in the SNc (*P* < 0.0001, Fig. 2D), compared to the control mice. Consistent with the behavioral results, FMT from PD patients led to a further loss of TH^+^ fibers in the striatum (the MPTP + PD FMT group *vs.* the MPTP group, *P* < 0.05, Fig. 2B) and TH^+^ somata in the SNc (the MPTP + PD FMT group *vs.* the MPTP group, *P* < 0.0001, Fig. 2D). In contrast, FMT from healthy human controls partially rescued TH^+^ fibers in the striatum (the MPTP + HC FMT group *vs.* the MPTP group, *P* < 0.0001, Fig. 2B) and TH^+^ somata in the SNc (the MPTP + PD FMT group *vs.* the MPTP group, *P* < 0.0001, Fig. 2D). Western blotting revealed similar results, with a parallel trend in alterations of TH expression in the four groups of mice (Fig. 2E-G). These findings indicated that fecal microbiota from PD patients may lead to dopaminergic neurodegeneration in MPTP-treated mice, but fecal microbiota from healthy human controls may protect against dopaminergic neuro-degeneration in MPTP-induced PD mice.

### FMT from healthy human controls ameliorated glial activation in both the striatum and SNc in MPTP-induced PD mice

**Figure 3.**
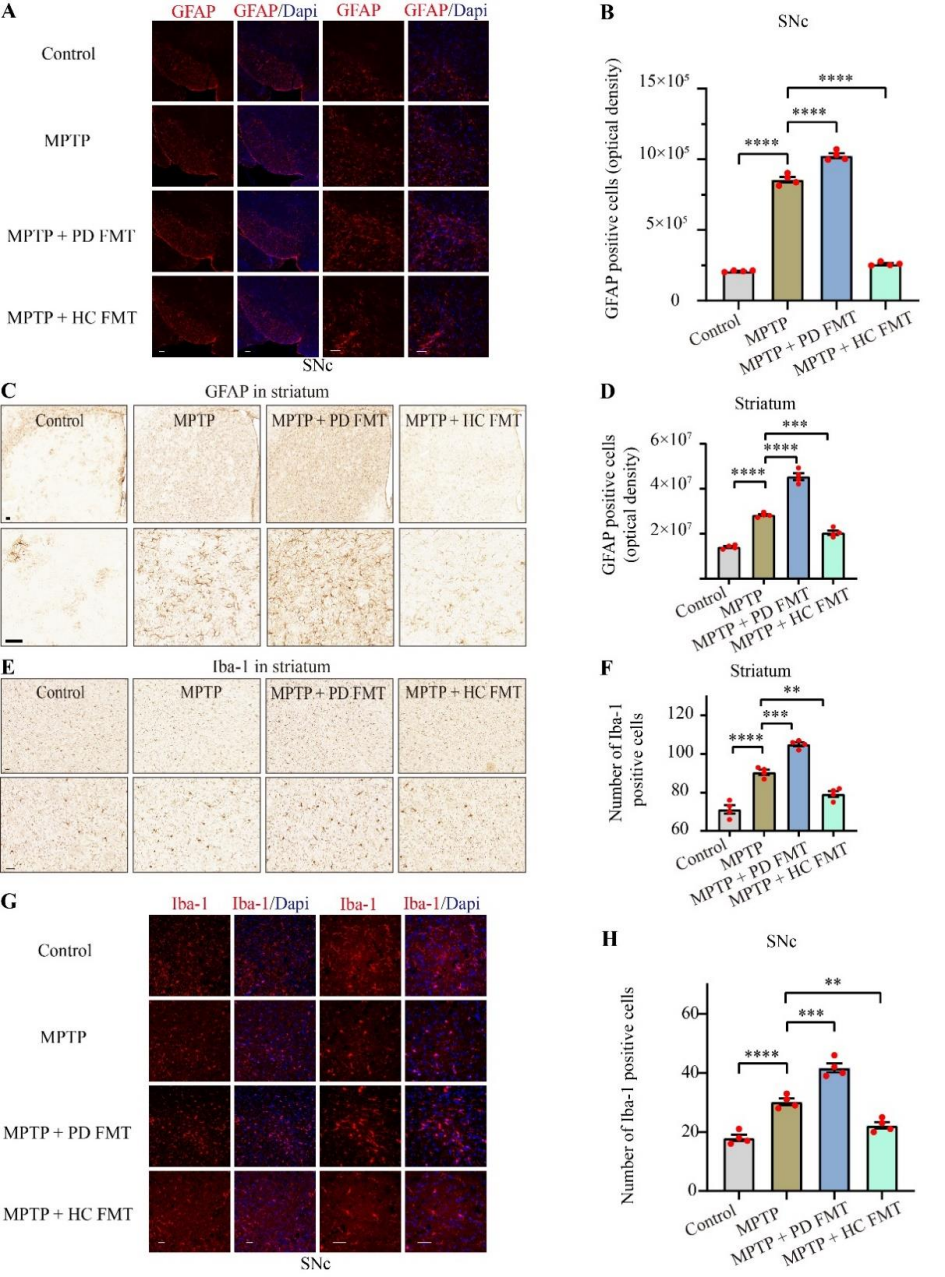
FMT from healthy humans alleviated glial activation in both the striatum and SNc in MPTP-treated mice. Representative images of GFAP^+^ (red) cells in the SNc (A) detected by immunofluorescence. Quantitative analysis of GFAP^+^ cells in the SNc (B). Representative images of GFAP^+^ cells in the striatum (C) and Iba-1^+^ cells in the striatum (E) stained by immunohistochemistry. Quantification of GFAP expression in the striatum (D). Quantification of Iba-1^+^ cells in the striatum (F). Representative images of Iba-1^+^ (red) cells in the SNc (G). Quantitative analysis of Iba-1^+^ cells in the SNc (H). All scale bars are 50 µm. Statistical analysis was performed using one-way ANOVA followed by Tukey test for multiple comparisons with n = 4, and error bars indicate mean ± SEM. Statistical significance was denoted by ** *P* < 0.01, *** *P* < 0.001, and **** *P* < 0.0001 (*P* values were adjusted with 6 tests).

Neuroinflammation in PD is characterized by nigrostriatal gliosis, including microgliosis and astrocytosis [41], which are often assessed by immunostaining for glial fibrillary acidic protein (GFAP) [42] and ionized calcium-binding adapter molecule 1 (Iba-1) [43], respectively. To assess the potential contribution of FMT from PD patients to glial-mediated neuroinflammation, we evaluated glial activation by immunostaining for GFAP and Iba-1 in both the SNc and striatum. As shown in Fig. 3, the MPTP-treated mice exhibited a marked increase in GFAP-positive cells in both the SNc (*P* < 0.0001, Fig. 3B) and striatum (*P* < 0.0001, Fig. 3D), compared to the control mice. Intriguingly, the MPTP + PD FMT mice showed a significant enhancement in GFAP^+^ cells in both the SNc (*P* < 0.0001, Fig. 3B) and striatum (*P* < 0.0001, Fig. 3D), compared to the MPTP group. Conversely, mice in the MPTP + HC FMT group showed a significant reduction in GFAP^+^ cells in both the SNc (*P* < 0.0001, Fig. 3B) and striatum (*P* < 0.001, Fig. 3D), compared to the MPTP group. Similarly, as seen in Fig. 3E-H, Iba-1^+^ cells in the nigrostriatal region of all four groups of mice exhibited a similar trend to GFAP^+^ cells. These results indicated that the gut microbiota from PD patients might exacerbate dopaminergic neurodegeneration by enhancing the activation of microglia and astrocytes, while the gut microbiota from healthy human controls might rescue dopaminergic neurons by inhibiting glial activation in MPTP-treated mice.

**Figure 4.**
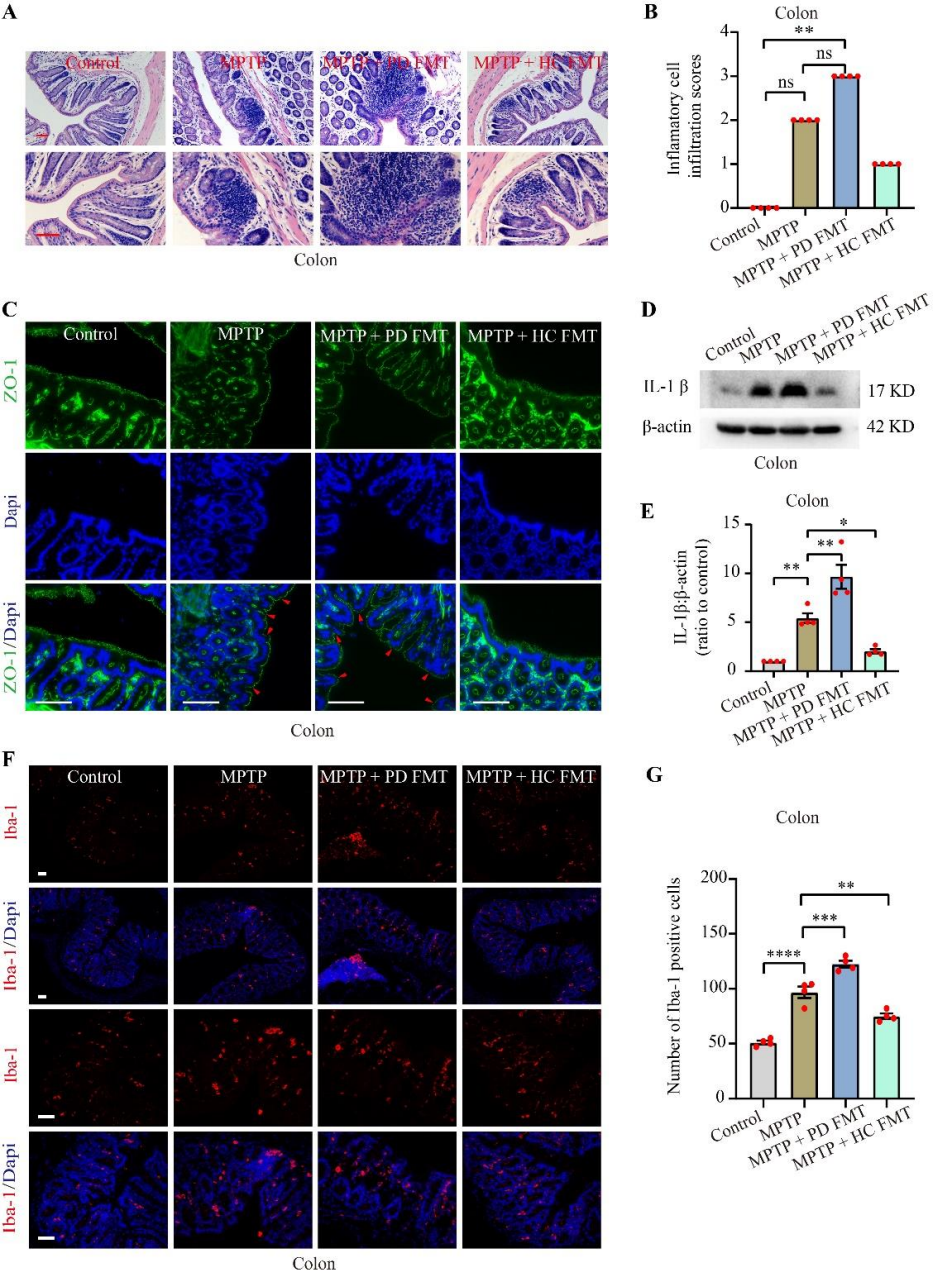
FMT from healthy humans moderated inflammation in the colon caused by MPTP. Representative images of inflammatory cell infiltration into the colon (A), as detected by H&E staining. Histologic scores of inflammatory cell infiltration in the colon (B). Representative images of ZO-1 (green) visualized by immunofluorescence in the colon (C), and the red arrowhead indicates disruption of the intestinal epithelial barrier. Representative western blot images showing IL-1β expression in the colon (D). Quantification of western blot bands indicating IL-1β expression in the colon (E). Representative images of Iba-1^+^ (red) cells in the colon (F), measured by immunofluorescence. Quantification of Iba-1^+^ cells in the colon (G). All scale bars are 50 µm. Statistical analysis was performed using one-way ANOVA followed by Tukey test for multiple comparisons for data with normal distribution (n = 4, error bars indicating mean ± SEM), except that histologic scores of inflammatory cell infiltration was analyzed using Kruskal-Wallis test followed by Dunn’s test due to the non-normal distributed data (n = 4, data presented as median (IQR)). Statistical significance was denoted by * *P* < 0.05, ** *P* < 0.01, *** *P* < 0.001, and **** *P* < 0.0001 (*P* values were adjusted with 6 tests). Abbreviation includes ns for no significance.

**Table 1:** OTUs and alpha-diversity.

	Groups				*P* values			
	Con	M	M+PF	M+HF	Con vs. M	M vs. M+PF	Con vs. M+PF	Con vs. M+HF
OTUs	657.1 ± 45.9	501.3 ± 59.4	380.7 ± 60.4	608 ± 34.5	0.1651	0.3626	0.0043**	0.9040
Chao1	662.7 ± 47.2	507.1 ± 61.1	383.6 ± 61.3	616.5 ± 36.0	0.1833	0.3644	0.0050**	0.9239
Shannon	7.01 ± 0.17	6.01 ± 0.21	4.79 ± 0.38	6.20 ± 0.18	0.0462*	0.0109*	<0.0001****	0.1316
Simpson	0.98 (0.02)	0.95 (0.05)	0.85 (0.20)	0.95 (0.07)	0.1442	0.3470	0.0002***	0.1112
Pielou	0.75 ± 0.01	0.68 ± 0.01	0.56 ± 0.03	0.67 ± 0.02	0.0668	0.0033**	<0.0001****	0.0541
Goods coverage	>0.99 ± 0.00	>0.99 ± 0.00	>0.99 ± 0.00	>0.99 ± 0.00	0.9593	0.6594	0.3703	0.8006

Statistical analysis was performed using one-way ANOVA followed by Tukey test for multiple comparisons to assess OTUs, Chao1, Shannon, Pielou, and Goods coverage (n = 7, mean ± SEM). For Simpson, Kruskal-Wallis test followed by Dunn’s test for multiple comparisons was used (n = 7, median (IQR)). Statistical significance was denoted as * *P* < 0.05, ** *P* < 0.01, *** *P* < 0.001, and **** *P* < 0.0001 (*P* values were adjusted with 6 tests in Tukey test and 5 tests in Dunn’s test). Abbreviations used include Con for Control, M for MPTP, M+PF for MPTP+PD FMT, and M+HF for MPTP+HC FMT.

### FMT from healthy human controls relieved inflammation induced by MPTP in the colon

Gastrointestinal disorders and inflammation commonly occur in PD patients [44]. To demonstrate the effect of FMT from PD patients and healthy human controls on the colon in MPTP-treated mice, we tested inflammation in the gut via H&E staining, immunofluorescence and western blotting. To evaluate inflammatory cell infiltration in colon, we analyzed the histologic scores of mice. While there was no statistically significant difference observed between the scores of the MPTP group and the control group, it is noteworthy that all mice in the MPTP group scored apparently higher than those of the control group (Fig. 4B). In addition, the scores were significantly increased in mice given FMT from PD patients (*P* < 0.01, Fig. 4B), compared to the control group. Although there was no statistically significant difference, FMT from healthy human controls markedly reduced the scores compared to the MPTP group (Fig. 4B). The tight junction protein zonula occludens protein 1 (ZO-1) is critical in maintaining the integrity of the intestinal mucosal barrier [24]. To evaluate the integrity of the colonic mucosal barrier, we used immunofluorescence staining to examine ZO-1 in mouse colons. Interestingly, we noted an apparent disruption in the intestinal mucosal barrier in MPTP-treated mice and in mice from the MPTP + PD FMT group (Fig. 4C, red arrowheads indicate disruption of the intestinal mucosal barrier), while the intestinal mucosal barrier remained intact in the MPTP + HC FMT group mice and the control group mice. Furthermore, expression of IL-1β was obviously increased in the colon of MPTP-treated mice (*P* < 0.01, Fig. 4E), compared the control group. Moreover, expression of IL-1β was even higher in MPTP + PD FMT group mice than in MPTP-treated mice (*P* < 0.01, Fig. 4E). However, expression of IL-1β was significantly suppressed in the MPTP + HC FMT group mice compared to the MPTP-treated mice (*P* < 0.05, Fig. 4E). The quantity of Iba-1+ cells demonstrated a comparable pattern to that of IL-1β expression in the colons (Fig. 4G). These results suggested that MPTP can disrupt the intestinal mucosal barrier and cause gut inflammation. The fecal microbiota from PD patients may promote damage, but the fecal microbiota from healthy controls might attenuate disruption and inflammation.

**Table 2:** Relative abundance of microbiota at the phylum level.

Taxonomic level	Relative abundance (%)				*P* values			
	Con	M	M+PF	M+HF	Con vs. M	M vs. M+PF	Con vs. M+PF	Con vs. M+HF
*Bacteroidota*	57.22 ± 5.00	38.03 ± 5.10	27.28 ± 4.22	44.11 ± 7.13	0.0887	0.5177	0.0038**	0.3481
*Firmicutes*	33.60 ± 4.31	38.31 ± 5.04	31.40 ± 4.82	42.30 ± 6.38	0.9174	0.7833	0.9904	0.6420
*Verrucomicrobiota*	2.57 (5.73)	0.88 (7.05)	36.59 (33.30)	7.51 (11.97)	>0.9999	0.0006***	0.0101*	>0.9999
*Desulfobacterota*	0.27 (1.52)	13.91 (19.12)	1.79 (4.9)	0.36 (0.75)	0.0005***	0.1997	0.4126	>0.9999
*Actinobacteriota*	1.66 ± 0.37	2.44 ± 0.71	1.63 ± 0.34	1.72 ± 0.13	0.5993	0.5740	>0.9999	0.9997

Statistical analysis was performed using one-way ANOVA followed by Tukey test for multiple comparisons to assess *Bacteroidota*, *Firmicutes*, *Desulfobacterota*, and *Actinobacteriota* (n = 7, mean ± SEM). For *Verrucomicrobiota*, Kruskal-Wallis test followed by Dunn’s test for multiple comparisons was used (n = 7, median (IQR)). Statistical significance was denoted as * *P* < 0.05, ** *P* < 0.01, and *** *P* < 0.001 (*P* values were adjusted with 6 tests, except for 5 tests in *Verrucomicrobiota*). Abbreviations used include Con for Control, M for MPTP, M+PF for MPTP+PD FMT, and M+HF for MPTP+HC FMT.

**Figure 5.**
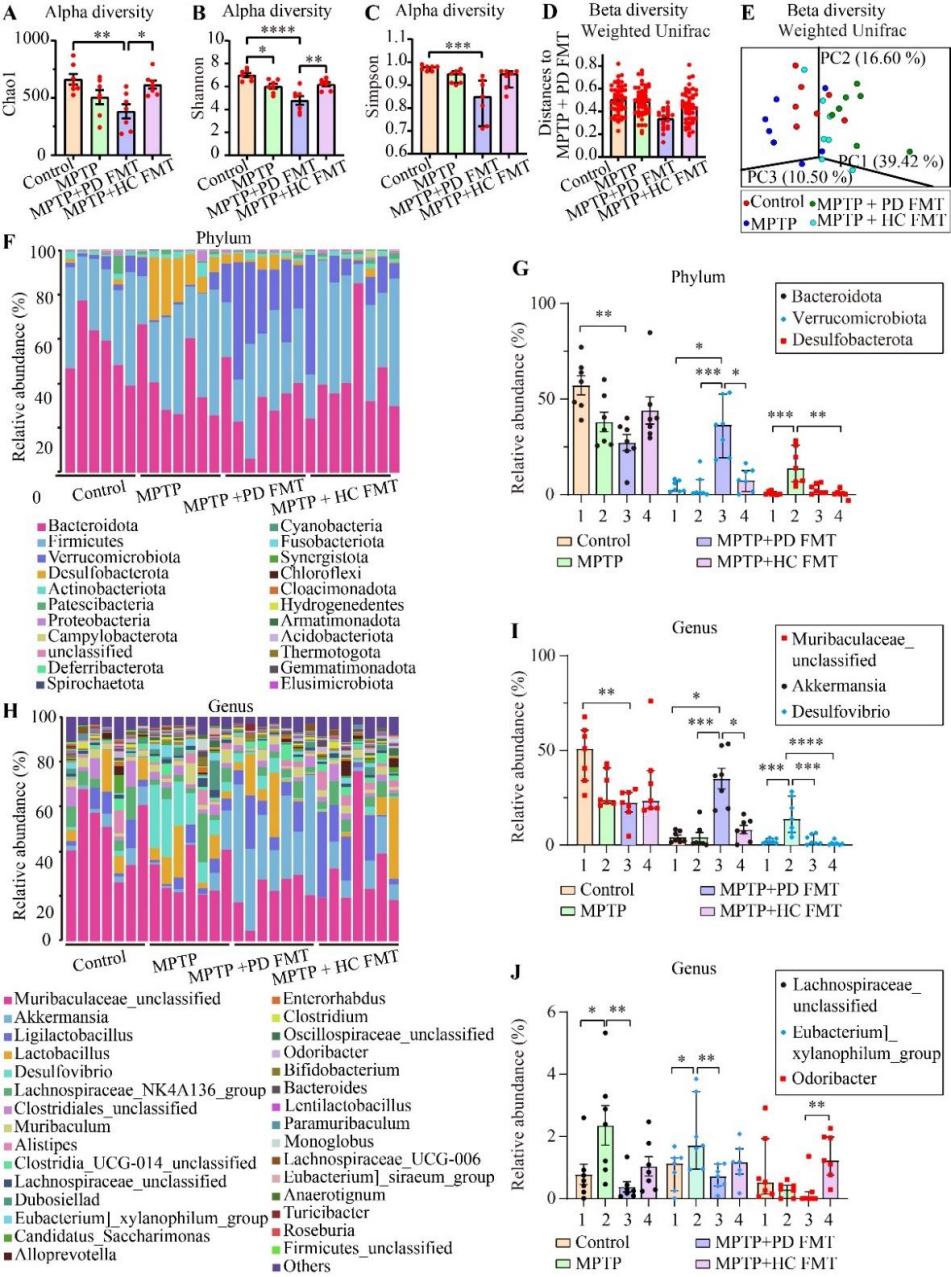
FMT from healthy humans reversed the MPTP-induced gut dysbacteriosis. Quantification of alpha diversity of gut microbiota, including the Chao1 (A), Shannon (B), and Simpson (C) indices. Quantification of the beta diversity of the gut microbiota, including weighted UniFrac analysis (ANOSIM, *R* = 0.4275, *P* = 0.0010) (D) and PCoA analysis (weighted UniFrac, *P* = 0.0010) (E). Relative abundance of gut microbiota at the phylum level (F). Quantification of relative abundance at the phylum level, including *Bacteroidota*, *Verrucomicrobioata*, and *Desulfobacterota* (G). Relative abundance of gut microbiota at the genus level (H). Quantification of relative abundance at the genus level, including *Muribaculaceae_unclassified, Akkermansia*, and *Desulfovibri*o (I). Quantification of relative abundance at the genus level, including *Lachnospiraceae_unclassified*, *[Eubacterium]_xylanophilum_group*, and *Odoribacter* (J). Statistical analysis was performed using one-way ANOVA followed by Tukey test for data with normal distribution (n = 7, error bars representing mean ± SEM), except that alpha diversity index Simpson, phylum *Verrucomicrobiota*, genus *Akkermansia*, genus *Desulfovibrio*, and genus *Odoribacter* was analyzed using Kruskal-Wallis test followed by Dunn’s test due to the non-normal distributed data (n = 7, data presented as median (IQR)). Statistical significance was denoted as * *P* < 0.05, ** *P* < 0.01, *** *P* < 0.001, and **** *P* < 0.0001 (*P* values were adjusted with 6 tests, except for 5 tests in *Verrucomicrobiota*, *Akkermansia*, and *[Eubacterium]_xylanophilum_group*).

### FMT from healthy human controls corrected the gut dysbacteriosis caused by MPTP

To investigate the alterations in gut microbiota in the four groups of mice, we performed 16S rDNA sequencing on mice feces. Detailed data concerning the full range of alpha diversity indices, including OTUs and the Chao1, Shannon, Simpson, Pielou, and Goods coverage indices for the four groups, with *P* values for multiple comparisons, were shown in Table 1. As demonstrated in Fig. 5 A-C, alpha diversity indices, including the Chao1 index, Shannon index and Simpson index, were significantly reduced in the MPTP + PD FMT group (*P* < 0.01, *P* < 0.0001, *P* < 0.001, respectively), compared to the control group. We also found an apparent decrease in the Shannon index in the MPTP group compared to the control group. However, higher indices, including Chao1 (*P* < 0.05) and Shannon (*P* < 0.01), were present in the MPTP + HC FMT group than in the MPTP + PD FMT group. Next, we analyzed the beta diversity of the microbiota using Principal Coordinates Analysis (PCoA) based on weighted UniFrac distances (*P* = 0.001, Fig. 5E) and illustrated the distinct microbiota communities among the 4 groups by ANOSIM analysis (*R* = 0.4275, *P* = 0.001, Fig. 5D). The microbiota community structures in the MPTP + PD FMT group exhibited significant differences compared to those in the control group, while there were no significant differences between the control group and the MPTP + HC FMT group. Table 2 and Table 3 showed detailed information on microbes at the phylum level (top 5 in abundance) and genus level (9 major breeds), including relative abundances and the *P* values for multiple comparisons. At the phylum level, the MPTP + PD FMT group was characterized by a significantly lower abundance of *Bacteroidota P* < 0.01, Fig. 5G) and a markedly higher abundance of *Verrucomicrobiota* (*P* < 0.05, Fig. 5G), relative to the control group. In addition, the MPTP group mice showed a significantly higher abundance of *Desulfobacterota* (*P* < 0.001, Fig. 5G), relative to the control group. At the genus level, the MPTP + PD FMT group displayed a remarkable increase in the abundance of *Akkermansia* (the MPTP + PD FMT group *vs.* the control group, *P* < 0.05, Fig. 5I), and an obvious decrease in both *unclassified Muribaculaceae* (the MPTP + PD FMT group *vs.* the control group, *P* < 0.01, Fig. 5I) and *Odoribacter* (the MPTP + PD FMT group *vs.* the MPTP + HC FMT group, *P* < 0.01, Fig. 5J). Meanwhile, the MPTP group showed a significant increase in *Desulfovibrio* abundance (the MPTP group *vs.* the control group, *P* < 0.001, Fig. 5I), and an obvious increase in both the *[Eubacterium]_xylanophilum_group* (the MPTP group *vs.* the MPTP + PD FMT group, *P* < 0.01, Fig. 5J) and *unclassified Lachnospiraceae* (the MPTP group *vs.* the control group, P < 0.05, Fig. 5J). To identify a potential biomarker of gut microbiota, we conducted linear discriminant analysis effect size (LEfse) analysis, which produced results (Supplementary Fig. 1C) matched those shown in Fig. 5E-J. Supplementary Fig. 1C showed the characteristic enriched microbiota in each group. We investigated the relationship of the microbial community structure among the 4 groups constructing a Venn diagram of the amount of amplicon sequence variants (ASVs) and a correlation heatmap at the genus level based on the Bray-Curtis distance (Supplementary Fig. 1A, B). The control group had the most characteristic ASVs (1296) while the MPTP + PD FMT group had the least (640). Moreover, the control group shared the most ASVs with the MPTP + HC FMT group (42.34%) and the least with the MPTP + PD FMT group (29.18%). At the genus level, the MPTP + HC FMT group was the most similar to the control, and the MPTP + PD FMT group showed the greatest separation from the control group (Supplementary Fig. 1).

### FMT from healthy human controls ameliorated the AMPK/SOD2 signaling pathway in both the colon and SNc in MPTP-treated mice

**Figure 6.**
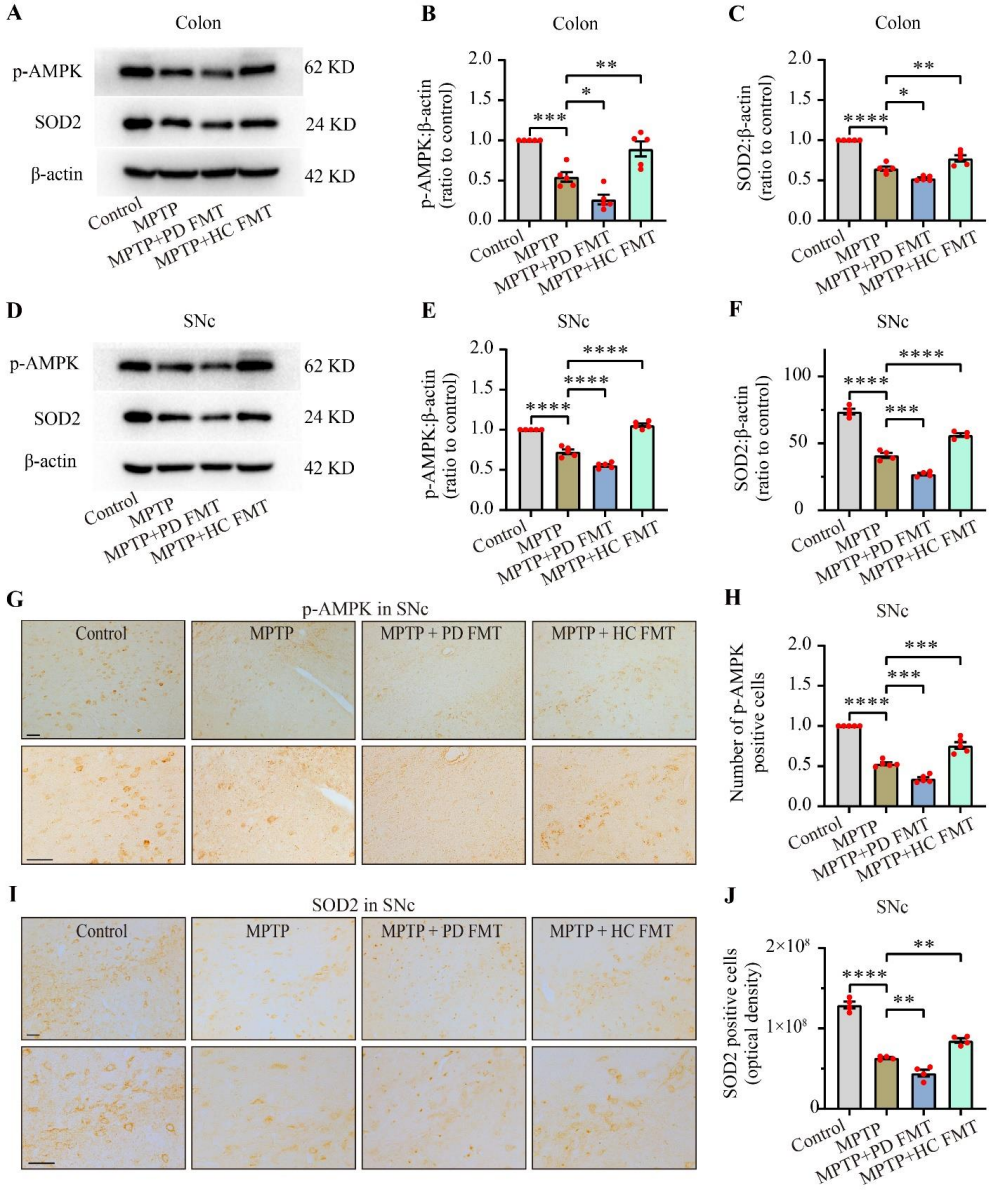
FMT from healthy humans restored MPTP-induced impairment of the p-AMPK/SOD2 pathway in both the colon and SNc. Representative western blot images showing p-AMPK and SOD2 expression in the colon (A) and SNc (D). Quantification of western blot bands indicating p-AMPK expression in the colon (B) and SNc (E). Quantification of western blot bands indicating SOD2 expression in the colon (C) and SNc (F). Representative images of p-AMPK (G) and SOD2 (I) in the SNc, measured by immunohistochemistry. Quantification of p-AMPK^+^ cells in the SNc (H). Quantification of SOD2 expression in the SNc (J). Scale bars are 50 µm. Statistical analysis was conducted using one-way ANOVA followed by Tukey test for multiple comparisons, with n = 5, and error bars indicate the mean ± SEM. Significant differences are indicated as * *P* < 0.05, ** *P* < 0.01, *** *P* < 0.001, and **** *P* < 0.0001 (*P* values were adjusted with 6 tests).

To test our hypothesis that fecal microbiota from PD patients or healthy human controls might regulate dopaminergic neurodegeneration via the AMPK/SOD2 signaling pathway, we used western blotting and immunohistochemistry analysis to detect the expression of phosphorylated AMPK (p-AMPK) and SOD2 protein in the colon and SNc of mice. As shown in Fig. 6, MPTP-treated mice exhibited obvious decreases in both p-AMPK (*P* < 0.001, Fig. 6B), and SOD2 (*P* < 0.0001, Fig. 6C) in the colon compared to the control group. MPTP + PD FMT mice showed even lower expression of p-AMPK (*P* < 0.05, Fig. 6B) and SOD2 (*P* < 0.05, Fig. 6C) in the colon compared to the MPTP group. In contrast, mice receiving FMT from healthy human controls exhibited a significant elevation in both p-AMPK (*P* < 0.01, Fig. 6B) and SOD2 (*P* < 0.01, Fig. 6C) in the colon compared to the MPTP group. Concomitant with the changes in p-AMPK and SOD2 in the colon, we observed a parallel trend in p-AMPK and SOD2 in the SNc (Fig. 6D-F). Immunohistochemistry staining of p-AMPK and SOD2 in the SNc further supported these findings (Fig. 6G-J).

**Figure 7.**
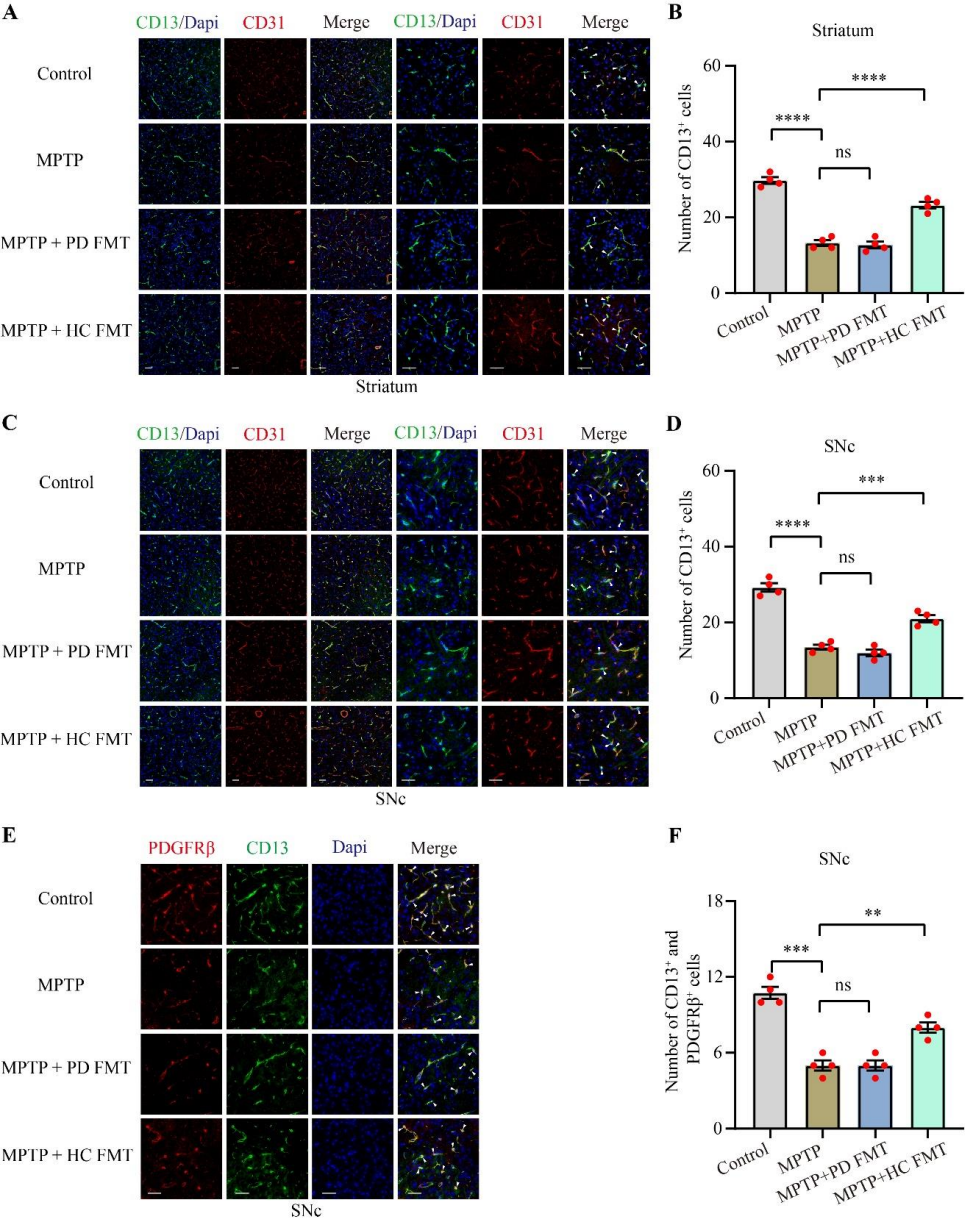
FMT from healthy human controls rescued the pericyte survival reduced by MPTP. Representative immunofluorescence staining images of CD13 (green) and CD31 (red) in the striatum (A). Quantification of CD13^+^ cells in the striatum (B). Representative immunofluorescence staining images of CD13 (green) and CD31 (red) in the SNc (C). Quantification of CD13^+^ cells in the SNc (D). Representative immunofluorescence staining images of CD13 (green) and PDGFRβ (red) in the SNc (E). Quantification of both CD13^+^ and PDGFRβ^+^ cells in the SNc (F). White arrow heads indicate pericytes. Scale bars are 50 µm. Statistical analysis was performed using one-way ANOVA followed by Tukey test for multiple comparisons, with n = 4, and error bars indicate the mean ± SEM. Statistical significance was denoted by * *P* < 0.05, ** *P* < 0.01, *** *P* < 0.001, and **** *P* < 0.0001 (*P* values were adjusted with 6 tests). Abbreviation includes ns for no significance.

**Table 3:** Relative abundance of microbiota at the genus level.

Taxonomic level	Relative abundance (%)				*P* values			
	Con	M	M+PF	M+HF	Con vs. M	M vs. M+PF	Con vs. M+PF	Con vs. M+HF
*Muribaculaceae_* *unclassified*	48.22 ± 5.70	29.68 ± 3.60	21.47 ± 3.25	32.68 ± 7.80	0.0987	0.7088	0.0093**	0.2037
*Akkermansia*	2.57 (5.73)	0.87 (7.05)	36.59 (33.31)	7.51 (11.96)	>0.9999	0.0006***	0.0101*	>0.9999
*Lactobacillus*	4.92 (9.66)	5.34 (9.70)	3.60 (17.50)	0.52 (8.42)	>0.9999	>0.9999	>0.9999	>0.9999
*Lachnospiraceae_* *NK4A136_group*	4.79 (8.56)	3.01 (4.14)	2.83 (2.82)	4.53 (5.26)	>0.9999	>0.9999	>0.9999	>0.9999
*Muribaculum*	2.85 ± 0.42	3.61 ± 1.13	2.70 ± 0.67	2.57 ± 0.40	0.8773	0.8097	0.9989	0.9926
*Desulfovibrio*	2.07 ± 0.43	15.39 ± 3.56	2.76 ± 0.99	0.91 ± 0.44	0.0002***	0.0004***	0.9936	0.9709
*[Eubacterium]_* *xylanophilum_group*	0.78 ± 0.32	2.36 ± 0.64	0.38 ± 0.17	1.04 ± 0.31	0.0491	0.0089**	0.9623	0.9946
*Lachnospiraceae_* *unclassified*	0.93 ± 0.23	2.07 ± 0.43	0.68 ± 0.15	1.13 ± 0.23	0.0398*	0.0095**	0.9226	0.9574
*Odoribacter*	0.53 (1.78)	0.30 (0.43)	0.00 (0.22)	1.23 (1.22)	>0.9999	>0.9999	0.2477	>0.9999

Microbiota at genus level, except for *Akkermansia*, *Desulfovibrio*, and *Odoribacter*, were analyzed by One-way ANOVA followed by Tukey test for multiple comparisons (n = 7, mean ± SEM). For the latter three taxa, Kruskal-Wallis test followed by Dunn’s test for multiple comparisons was used (n = 7, median (IQR)). Statistical significance was denoted as * *P* < 0.05, ** *P* < 0.01, and *** *P* < 0.001 (*P* values were adjusted with 6 tests, except for 5 tests in *Akkermansia*, and *[Eubacterium]_ xylanophilum_group*). Abbreviations used include Con for Control, M for MPTP, M+PF for MPTP+PD FMT, and M+HF for MPTP+HC FMT.

These data suggested that MPTP treatment inhibited the AMPK/SOD2 signaling pathway in both the colon and the SNc in mice and that FMT from PD patients further suppressed the pathway. In contrast, FMT from healthy human controls restored the pathway.

### FMT from healthy human controls improved the survival of pericytes injured by MPTP

Disruption of the BBB is involved in the development of neurodegenerative diseases, including PD [45]. Pericytes, which lack specific markers, are essential components of the BBB. Multiple markers, such as CD13 and PDGFRβ, are commonly used to label pericytes [46]. To explore alterations in pericytes in MPTP-treated mice and explore the influence of the gut microbiota from healthy human controls or PD patients on pericytes, we used immunofluorescence to examine CD13, PDGFRβ, along with the vascular endothelial cell marker CD31 (Fig. 7). Compared to the control mice, MPTP-treated mice showed a significant reduction in the number of CD13^+^ cells in both the striatum (*P* < 0.0001, Fig. 7B) and SNc (*P* < 0.0001, Fig. 7D). However, mice in the MPTP + HC FMT group exhibited a significant elevation in pericytes in the striatum (*P* < 0.001, Fig. 7B) and SNc (*P* < 0.001, Fig. 7D), compared to the MPTP group. Moreover, MPTP-treated mice showed an apparent loss of CD13^+^ and PDGFRβ^+^ cells (*P* < 0.001, Fig. 7F), relative to the control group. Although MPTP + PD FMT mice did not exhibit significant alterations in pericyte markers in either the striatum (Fig. 7B) or SNc (Fig. 7D, F) relative to MPTP-treated mice, MPTP + HC FMT mice showed significantly more CD13^+^ and PDGFRβ^+^ cells in the SNc (the MPTP + HC FMT group vs. the MPTP group, P < 0.01, Fig. 7F). These results indicated that MPTP may damage pericytes, but gut microbiota from healthy controls may play a potential protective role in pericytes in MPTP-injected mice.

## DISCUSSION

Several recent studies have suggested that alterations in the gut microbiota participate in modulating neuroinflammation in PD [17, 18, 47]. In addition, it has been reported that mitochondria might play a crucial role in modulating immunoreactions, linking neuro-degeneration to neuroinflammation [48]. Here, we investigated whether mitochondrial antioxidative capacity is involved in inflammation regulated by the gut microbiota. Our study showed that FMT from PD patients or healthy controls to MPTP-treated mice can regulate the neurotoxic effects of MPTP. We described four main findings. First, FMT from PD patients to MPTP-treated mice aggravated the neurotoxic effects of MPTP, which may be attributed to an increase in *Akkermansia*. Second, FMT from healthy human controls to mice alleviated MPTP-mediated neurotoxicity, which may be due to the correction of gut dysbacteriosis. Third, FMT from healthy humans to MPTP-treated mice attenuated neurotoxicity by upregulating the AMPK/SOD2 signaling pathway. Finally, MPTP induced a loss of nigrostriatal pericytes, which was partially prevented by transplantation of fecal microbiota from healthy human controls to MPTP-treated mice.

**Figure 8.**
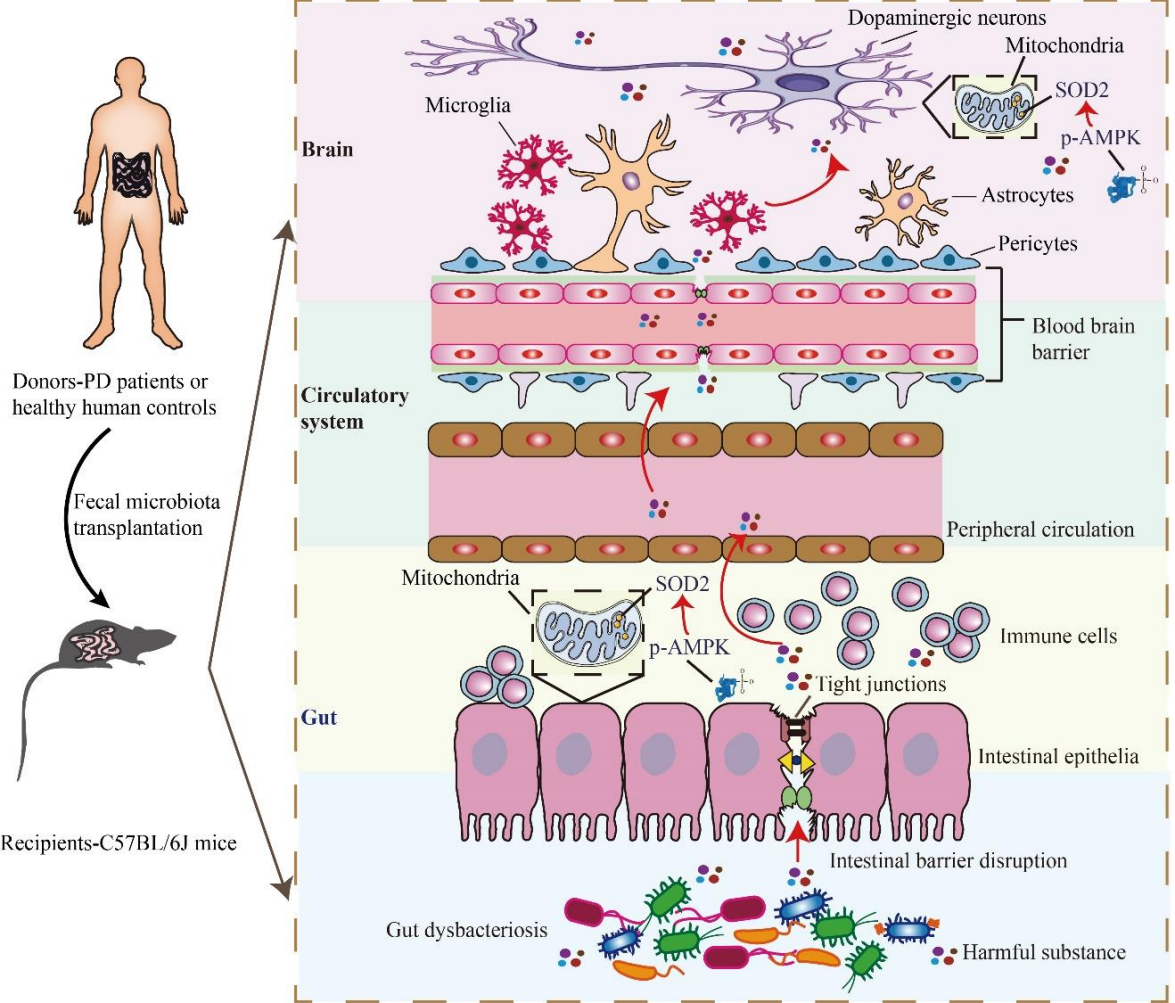
Schematic illustration of the underlying mechanism. FMT from healthy human controls revers gut dysbacteriosis, improves colonic tight junction integrity, alleviates colonic inflammation, rescues dopaminergic neurodegeneration, inhibits nigrostriatal glial activation, prevents loss of nigrostriatal pericytes and restores the AMPK/SOD2 pathway in MPTP-treated mice.

In the present study, FMT from PD patients to mice significantly aggravated motor impairments in MPTP-treated mice, while FMT from healthy human controls remarkably prevented motor dysfunction (Fig. 1 and Fig. 2). Consistently, previous studies have demonstrated that FMT from healthy control mice relieved motor impairments in MPTP/rotenone-induced PD mice [18, 19]. Our study successfully established the PD mouse model as demonstrated by significant loss of TH^+^ fibers in the striatum and TH^+^ somas in the SNc following MPTP administration. However, compared to MPTP-treated mice, the mice receiving fecal microbiota from healthy human controls showed less loss of TH^+^ neurons, consistent with previous reports [18, 49]. Of note, the mice given fecal microbiota from PD patients exhibited even worse loss of TH^+^ neurons, corroborating previous findings [17]. Taken together, the fecal microbiota from PD patients may aggravate motor disorders in MPTP-treated mice by increasing the loss of TH^+^ neurons, whereas the fecal microbiota from healthy human controls may alleviate these disorders by reducing the loss of TH^+^ neurons in mice.

Mitochondrial dysfunction, oxidative damage, and neuroinflammation mediated by glial activation are key contributors to dopaminergic neurodegeneration in PD [9, 50]. In the current study, MPTP-induced glial activation in the striatum and SNc was significantly attenuated by FMT from healthy human controls, indicating that the gut microbiota from healthy human controls may suppress inflammation through the gut-brain axis in MPTP-treated mice. Interestingly, oral administration of fecal microbiota from PD patients significantly increased nigrostriatal glial activation in MPTP-treated mice, suggesting that some bacteria or their metabolites in the feces of PD patients may stimulate glial activation, or that FMT from PD patients may enhance proinflammatory responses by amplifying gut dysbacteriosis. Gut dysbacteriosis commonly occurs in PD and PD animal models and is related to inflammation in the gut [51, 52]. Furthermore, we investigated the effects of FMT on gut inflammation and found MPTP-treated mice had more inflammatory cell infiltration, significant destruction of the intestinal mucosal barrier and higher expression of IL-1βin the colon compared to the control mice. Interestingly, FMT from PD patients to mice amplified these impacts, while FMT from healthy human controls prevented them (Fig. 4). Collectively, the results showed that FMT from humans to mice can regulate inflammation in both the brain and gut, suggesting that alterations in the gut microbiota may be involved in the regulation of dopaminergic neurodegeneration in MPTP-treated mice.

To further investigate the potential role of alterations in gut microbiota in the phenotypes observed in the four groups of mice, we used 16S rDNA sequencing to test the fecal microbiota. Our results confirmed a state of gut dysbacteriosis in PD fecal microbiota recipient mice, whose alpha diversity indices were significantly lower than those of the control mice (Fig. 5 and Table 1). Additionally, the beta diversity indices of MPTP + PD FMT mice were significantly different from those of the control mice, indicating a distinct composition and structure of the gut microbiota of the MPTP + PD FMT group (Fig. 5). Furthermore, the control group exhibited the furthest distance from the MPTP + PD FMT group at the genus level, sharing the least ASVs with the MPTP + PD FMT group (Supplementary Fig. 1). Several studies have reported a higher abundance of *Akkermansia* in PD patients compared to healthy controls [16, 53], although *Akkermansia* is generally thought to be a probiotic bacterium owing to its protective effects on gut barrier function and anti-inflammatory properties [54]. Nevertheless, an increase in *Akkermansia* has also been linked to reduced mucus and decreased resistance to a specific pathogen in mice [55]. Here, we observed significantly increased *Akkermansia* in PD fecal microbiota recipient mice compared to the control mice and MPTP-treated mice. Such results are consistent with previous studies that used toxin-induced PD animal models [19, 47, 53]. Collectively, gut dysbacteriosis, especially abnormally enriched *Akkermansia*, may enhance the disruption of gut barriers and promote inflammation in both the gut and brain of mice in the MPTP + PD FMT group. However, some studies found a different trend in *Akkermansia* abundance in PD mice [49, 56], which could be attributed to differences in intervention, sampling time, animal ages and species.

Acute administration of hydrogen sulfide (H_2_S) into airway can induce cytochrome oxidase inhibition in the animal brain, impairing mitochondrial function [57]. However, H_2_S exhibits a dual effect, being cytoprotective at low concentrations but toxic at high concentrations [58]. *Desulfovibrio*, one of the main H_2_S-producing gut bacteria [59], is known to be associated with PD due to its elevated abundance [60]. Strikingly, we found that *Desulfovibrio* was significantly enriched in MPTP-treated mice, which is consistent with previous findings showing that rotenone-induced PD mice also had a significantly higher abundance of *Desulfovibrio* [19]. In addition, a reduction in *Muribaculaceae* was reported in PD animal models [61], which is in agreement with the significant reduction of *Bacteroidota*, the phylum containing the *Muribaculaceae* genus, in both PD patients [62] and PD animal models [63]. Interestingly, we observed a notable decline in *Bacteroidota* and unclassified *Muribaculaceae* abundance (Fig. 5, Table 2, and Table 3). Although an increase in *[Eubacterium]_xylanophilum_group* has been linked to an autism animal model [64], but no association with PD has been reported. Importantly, our data showed a notable increase in the abundance of *[Eubacterium] _xylanophilum_group* in MPTP-treated mice, despite only accounting for approximately 2% of the total fecal microbiota (Fig. 5, Table 2, and Table 3). The reports on the alterations in *Odoribacter* abundance in PD patients have been inconsistent [65]. Here, we noticed a significant increase in *Odoribacter* abundance in mice receiving FMT from healthy human controls. Additionally, we found that MPTP-treated mice had a higher abundance of unclassified *Lachnospiraceae* than the control mice. We further verified the presence of gut dysbacteriosis in both MPTP-treated mice and PD fecal microbiota recipient mice, which were reversed by FMT from healthy human controls (Fig. 5, Table 1, Table 2, Table 3, and Supplementary Fig. 1). It is worth noting that the most significant alteration in MPTP-treated mice was the enrichment of *Desulfovibrio*, and the most significant alteration in PD patient fecal microbiota recipient mice was an increase in *Akkermansia*. Taken together, these gut microbiota alterations could underlie the different phenotypes of the four groups of mice. Nevertheless, more studies are needed to explore whether *Desulfovibrio* or *Akkermansia* alone could play a crucial role in the proinflammatory effects in PD.

The precise mechanisms underlying the communication between the gut microbiota and the brain remain elusive. Nonetheless, emerging research suggests that microbiota-derived metabolites may act as mediators in the microbiota-gut-brain axis. For instance, a decrease in short-chain fatty acids (SCFAs) in feces has been implicated in the development of PD [66], and administration of SCFAs has been shown to suppresses the secretion of proinflammatory cytokines by activating microglia-like cells [67]. Conversely, trimethylamine-N-oxide may enhance inflammation and oxidative stress [68]. In addition, N^6^-carboxymethyllysine may promote mitochondrial impairment and oxidative stress in microglia [69]. Lipopolysaccharide (LPS) appears to play a key role in the communication between the gut and the brain, as it has a remarkable proinflammatory effect [70]. Interestingly, L. plantarum DP189 was found to slow neurodegeneration via inhibition of oxidative stress, reduction of the proinflammatory response, and modification of the gut microbiota in mice [71]. Overall, this evidence indicates that the gut microbiota likely modulates inflammation by regulating mitochondrial susceptibility to oxidation. In our study, we observed lower expression of p-AMPK and SOD2 in the colon and nigrostriatal regions of MPTP-treated mice compared to the control mice, while higher expression of p-AMPK and SOD2 was detected in the same tissues of mice receiving fecal microbiota from healthy human controls, as opposed to MPTP-treated mice. Strikingly, even lower expression levels of p-AMPK and SOD2 in the same tissues were detected in mice given fecal microbiota from PD patients compared to MPTP-treated mice (Fig. 6). These results indicate that FMT from PD patients enhanced the MPTP-induced inhibition of the AMPK/SOD2 signaling pathway, while FMT from healthy human controls partially reversed the impact by upregulating this pathway. However, more studies are required to determine whether *Desulfovibrio* or *Akkermansia* directly or indirectly regulate the AMPK/SOD2 signaling pathway.

It has been documented that pericyte deficiency is sufficient to significantly increase the permeability of the BBB and cause damage to neurons [72]. Furthermore, nigrostriatal vascular leakage has been reported in MPTP-treated mice [73]. To the best of our knowledge, alterations in nigrostriatal pericytes in MPTP-induced PD mouse models have not yet been reported. Of note, we found a substantial reduction in nigrostriatal pericytes in the MPTP-treated mice (Fig. 7). Additionally, the loss of pericytes was partially restored by the fecal microbiota from healthy human controls, indicating that correction of gut dysbacteriosis may reduce MPTP-induced pericyte loss. The survival of pericytes was unaffected by the fecal microbiota from PD patients compared to MPTP-treated mice. In contrast to our findings, a notable increase in pericytes was observed in the lesioned striatum of 6-hydroxydopamine (6-OHDA)-induced PD mice [74]; however, alterations in pericytes in the SNc of 6-OHDA mice were not investigated. 6-OHDA is commonly administered to the striatum via stereotaxic injection due to BBB's impermeability to the compound [75]. This injection method causes unavoidable injury resulting from the needle passing through the brain tissue, such as hemorrhage. As a result of hemorrhage, some pericyte-responsive material might enter the brain tissue, potentially leading to pericyte proliferation. In contrast to Padel’s study, we established a PD mouse model by intraperitoneally injecting MPTP without causing direct brain tissue injury. As a result, the damage generated by the stereotaxic injection itself could partially explain the discrepancy in pericyte changes between our findings and the previous results [74] Due to its inability to freely cross the BBB and cytomembrane, 6-OHDA is mainly distributed around the injection site [76], and there is little evidence suggesting that stereotactic 6-OHDA injection can create significant alterations in the gut microbiota in PD animal models. Unlike 6-OHDA, intraperitoneal injection of MPTP can induce marked gut dysbacteriosis [33, 49, 71]. Strikingly, FMT from control mice enhanced pericyte coverage in a spinal cord injury mouse model [77]. Thus, the presence of gut dysbacteriosis may also partly account for the discrepancy in pericyte-related findings between our study and that of Padel [74]. Therefore, we hypothesize that MPTP-induced gut dysbacteriosis may facilitate the entry of harmful microbiota-derived substances into the brain, resulting in pericyte damage, BBB breakdown, reduced neuronal mitochondrial antioxidant capacity, and increased neuroinflammation and dopaminergic neurodegeneration. These harmful substances may include specific microbiota-derived metabolites, such as SCFAs, or toxic substances released by bacteria, such as LPS. This hypothesis suggests a potential mechanism by which gut dysbacteriosis can contribute to the pathogenesis of PD (Fig. 8). Hence, further studies are required to better understand the specific harmful substance derived from gut microbiota and their effects on pericytes as well as neurons in PD.

Our findings differ from previous studies in three important aspects. First, we used a different animal model compared to prior studies on the topic [17-19]. It has been reported that FMT from PD patients can significantly worsen motor dysfunction in α-synuclein-overexpressing (ASO) mice compared to genotype-matched recipient mice receiving FMT from healthy human controls [17], but these findings only apply to the small portion of hereditary PD cases. Our study, on the other hand, found an amplification effect of the gut microbiota from PD patients on motor impairment in MPTP-treated mice, the most commonly used PD animal model that can mimic sporadic PD to some extent. Hence, our findings raise the possibility that the PD fecal microbiota contributes to the progression of PD. Second, we used FMT from humans to mice, rather than from mice to mice, as done in previous similar studies [18, 19]. Prior research has shown that FMT from healthy control mice exerted a neuroprotective effect in neurotoxin-induced PD mice [18, 19]. Our investigation not only revealed the potential effects of the fecal microbiota of PD patients on the development of PD but also further verified the beneficial effects of the fecal microbiota of healthy human controls on PD development. Finally, we investigated the role of mitochondrial antioxidative capacity in the crosstalk between gut microbiota and MPTP-induced inflammation. The relationship between mitochondria and fecal microbiota in neurotoxin-induced PD mice remains largely unknown. However, butyrate, a type of SCFA, can increase mitochondrial inner membrane proton leakage and decrease ROS [78], suggesting that the gut microbiota can regulate mitochondrial antioxidant activity via bacterial metabolites. Recently, it was reported that probiotic administration helped repair 6-OHDA-induced motor deficits by enhancing mitochondrial function in PD rats [79]. Given the high energy requirements of dopaminergic neurons, their mitochondria produce a large amount of ROS, which are constantly eliminated through antioxidant defenses, such as SOD2 [80]. Furthermore, the neurotoxicity of MPTP was significantly reduced in SOD2-overexpressing mice compared to wild-type mice [81]. AMPK activation has been reported to decrease mitochondrial ROS by upregulating SOD2 in a diabetes animal model [82]. Interestingly, our findings showed that FMT from PD patients further reduced protein expression levels of p-AMPK and SOD2 in MPTP-treated mice, while FMT from healthy human controls partially reversed this effect. Collectively, previous studies and the current study show a close link between gut microbiota and mitochondrial antioxidative capacity in PD mice.

### Conclusions

Our findings demonstrated that FMT from PD patients amplified the neurotoxicity of MPTP in mice, likely by inhibiting the AMPK/SOD2 pathway and aggravating gut dysbacteriosis, while FMT from healthy human controls alleviated this neurotoxicity by upregulating the AMPK/SOD2 pathway and adjusting gut dysbacteriosis. Importantly, we found that MPTP can induce remarkable loss of nigrostriatal pericytes, whereas FMT from healthy human controls partially prevented this loss of pericytes. Our results suggest that the gut microbiota from healthy human controls may regulate mitochondrial oxidation resistance to neuroinflammation and rescue nigrostriatal pericyte loss and BBB disruption mediated by MPTP in PD mice. Overall, our study underscores the crucial role of gut microbiota in the pathogenesis of PD and proposes that interventions aimed at manipulating the gut microbiota may hold great potential as an effective therapeutic approach for treating this debilitating disease. Nevertheless, more studies are required to further understand the specific bacteria or metabolites that contribute to neurodegeneration and elucidate their functions in PD.

## Supplementary Materials

The Supplementary data can be found online at: www.aginganddisease.org/EN/10.14336/AD.2023.0309.
