# Supplementary material for: Healthy Human Fecal Microbiota Transplantation into Mice Attenuates MPTP-Induced Neurotoxicity via AMPK/SOD2 Pathway
Source: Aging Dis. 2023 Dec 1;14(6):2193–214. doi: 10.14336/AD.2023.0309 (PMC10676800; doi:10.14336/AD.2023.0309)
Supplement: Supplementary file 1 — The Supplementary data can be found online at: www.aginganddisease.org/EN/10.14336/AD.2023.0309. [file AD-14-6-2193-s.pdf]

SUPPLEMENTARY DATA

Healthy Human Fecal Microbiota Transplantation into Mice Attenuates MPTP-Induced Neurotoxicity *via* AMPK/SOD2pathway

Zhenchao Xie[1#], Mahui Zhang[1#], Yuqi Luo[1#], Dana Jin[2], Xingfang Guo[1], Wanlin Yang[1], Jialing Zheng[1], Hongfei Zhang[3], Lu Zhang[4], Chao Deng[5], Wenhua Zheng[6], Eng-King Tan[7], Kunlin Jin[8], Shuzhen Zhu[1*], Qing Wang[1*]

© 2023. Xie Z et al. Published online at http://www.aginganddisease.org/EN/10.14336/AD.2023.0309

SUPPLEMENTARY DATA

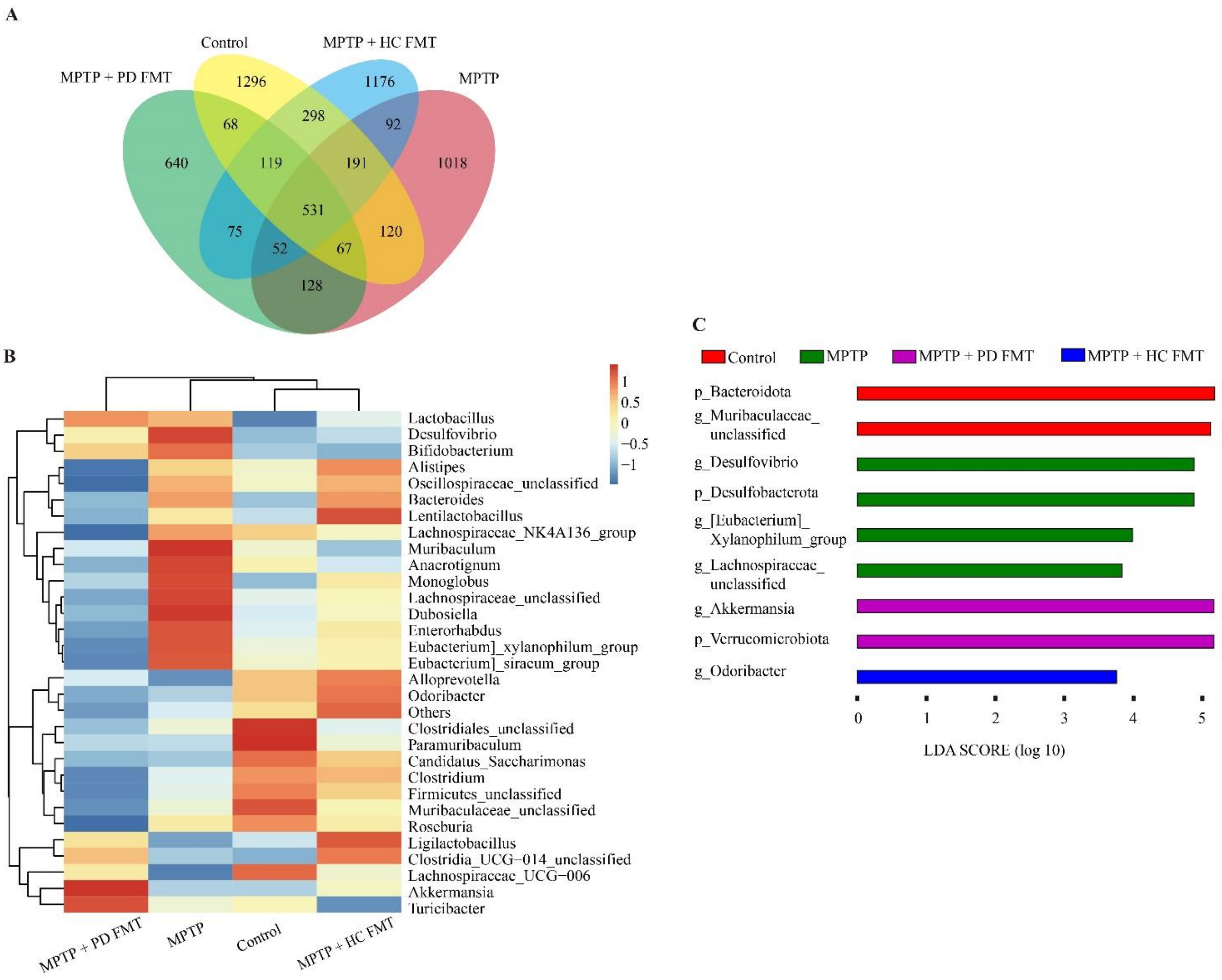

Supplementary Figure 1. Graphs illustrating the alterations in gut microbiota in mice in the 4 groups. (A) Venn diagram illustrating the distribution of gut microbiota features among the four groups. The numbers inside each circle indicate the number of unique ASVs identified for each group. (B) Heatmaps depicting correlation analysis of the relative abundance of gut microbiota at the genus level among the four groups, based on the Bray−Curtis distance metric. (C) Linear discriminant analysis effect size (LEfSe) plot of gut microbiota at the phylum and genus levels, revealing significant differences among the four groups. Phyla and genera are abbreviated as p_ and g_, respectively.

© 2023. Xie Z et al. Published online at http://www.aginganddisease.org/EN/10.14336/AD.2023.0309
